# Multifunctional Operation of an Organic Device with Three-Dimensional Architecture

**DOI:** 10.3390/ma12081357

**Published:** 2019-04-25

**Authors:** Giuseppe Tarabella, Simone Luigi Marasso, Valentina Bertana, Davide Vurro, Pasquale D’Angelo, Salvatore Iannotta, Matteo Cocuzza

**Affiliations:** 1 Institute of Materials for Electronics and Magnetism, IMEM-CNR, Parco Area delle Scienze 37/A, 43124 Parma, Italy; g.tarabella@camlintechnologies.com (G.T.); simone.marasso@polito.it (S.L.M.); davide.vurro@imem.cnr.it (D.V.); salvatore.iannotta@imem.cnr.it (S.I.); matteo.cocuzza@infm.polito.it (M.C.); 2 Camlin Italy Srl, Strada Budellungo 2, 43123 Parma, Italy; 3 Chilab, Materials and Microsystems Laboratory, Department of Applied Science and Technology (DISAT), Polytechnic of Turin, Via Lungo Piazza d’Armi 6, 10034 Chivasso (Turin), Italy; valentina.bertana@polito.it

**Keywords:** 3D-printed electronics, biosensor, organic electrochemical transistor, multifunctional operation

## Abstract

This work aims to show the feasibility of an innovative approach for the manufacturing of organic-based devices with a true three-dimensional and customizable structure that is made possible by plastic templates, fabricated by additive manufacturing methods, and coated by conducting organic thin films. Specifically, a three-dimensional prototype based on a polyamide structure covered by poly(3,4-ethylenedioxythiophene):polystyrene sulfonate (PEDOT:PSS) using the dip-coating technique demonstrated a multifunctional character. The prototype is indeed able to operate both as a three-terminal device showing the typical response of organic electrochemical transistors (OECTs), with a higher amplification performance with respect to planar (2D) all-PEDOT:PSS OECTs, and as a two-terminal device able to efficiently implement a resistive sensing of water vaporization and perspiration, showing performances at least comparable to that of state-of-art resistive humidity sensors based on pristine PEDOT:PSS. To our knowledge, this is the first reported proof-of-concept of a true 3D structured OECT, obtained by exploiting a Selective laser sintering approach that, though simple in terms of 3D layout, paves the way for the integration of sensors based on OECTs into three-dimensional objects in various application areas.

## 1. Introduction

The concept of three-dimensionality in the context of organic electronics applies to technological solutions implemented via the integration of organic-based devices [1] and extends to items fabricated using specific manufacturing methods [2], such as three-dimensional (3D) printing or additive manufacturing (AM). AM represents an emerging technique for the development of 3D-printed organic devices. It exploits a bottom-up approach through the layer-by-layer manufacturing of three-dimensional physical objects from a digital model; hence, the third dimension is achieved through the stacked deposition of layers.

Although the device prototypes proposed in the literature respond to specific requirements of large-area effectiveness [3], the extent of the third dimension along the out-of-plane axis is often very limited with respect to the in-plane device linear dimensions [4,5]. This is an important aspect, as the three-dimensional extent of devices can lead to new solutions and integration, offering several advantages in many fields of applications. For instance, the 3D conceptualization of device geometries enables the implementation of sensors for wearable electronics [6] and energy harvesting for building integration [7], but also biomedical devices for medicine [8]. The latter, in particular, benefited from the development of bioelectronics based on organic conductors, a class of materials showing peculiar properties of biocompatibility and conformability along with mixed ionic-electronic charge transport. Neural interfaces [9], neuromorphic devices [10] and biosensors [11] are just some of the applications based on devices made of conductive organics. Multifunctionality is another prerogative of some organic-based devices that are capable of adding at least another function to their intrinsic functionality, such as organic electrochemical transistors (OECTs) [12,13,14]. OECTs are very effective amplifiers of ionic signals that have been originally used as biosensors [15], but have shown great potentialities in neuromorphic applications [16,17] or as Lab-on-Chip platforms [18]. Combining the extremely interesting and versatile sensing characteristics of the OECTs with the 3D freedom of form, design, and structuring typical of AM, is a promising approach to obtain the next-generation devices. The basic idea is to transform the OECTs, belonging to the vast family of devices manufactured through planar thin-film (2D) technologies typical of the technological domain of microelectronics, in a device with similar (or possibly improved) performance, but also structured in the third dimension, e.g., for a direct transformation or integration of the sensing device into three-dimensional objects of common use (buttons, glasses, integrated into levers and controls in the passenger compartment of a car, integrated in sports gear, and so on). The exploitation of this approach could also potentially produce performance advantages, e.g., in terms of sensitivity [19].

In this work, an easy and cost-effective approach to fabricate a multifunctional truly 3D-printed organic device based on poly(3,4-ethylenedioxythiophene):polystyrene sulfonate (PEDOT:PSS), which is a diphasic organic conductor composed of conductive PEDOT clusters surrounded by a hydrophilic and insulating PSS phase, is reported. The proposed prototype shows OECT and resistive sensor responses. The device is fabricated by an approach consisting in coating a 3D structure made of polyamide and printed by commercially available selective laser sintering (SLS) 3D printer. The typical OECT operation demonstrated by the proof of concept prototype is assisted by the high surface area-to-volume ratio of PEDOT:PSS films as a consequence of the irregular surface micromorphology of the as-coated SLS prototype. This particular feature results in a marked sensitiveness to water vaporization upon operating the device as a resistive sensor (i.e., in a two-terminal configuration) next to liquid surfaces, as confirmed by test measurements carried out in presence of a phosphate buffer saline (PBS) solution at different concentrations in deionized (DI) water. The synergistic effect between the substrate porosity and the documented PEDOT:PSS humidity sensitiveness [20] is exploited to detect the evaporation generated by perspiration.

## 2. Materials and Methods

**Figure 1:**
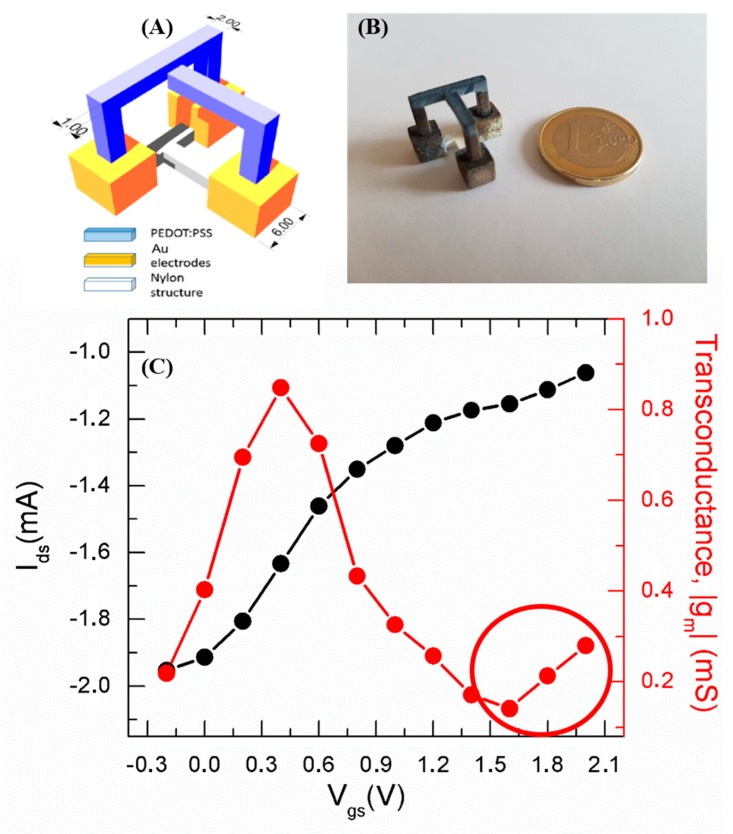
(**A**) Scheme of the designed 3D device (units are expressed in mm); (**B**) optical image of the fabricated 3D device; (**C**) organic electrochemical transistor (OECT) operation: transfer curve (black line-symbols) and related transconductance curve (red line-symbols) recorded by sweeping V_gs_ between −0.2 V and 2 V.

The 3D structure was fabricated using a SnowWhite SLS 3D printer (from Sharebot Srl, Nibionno, Italy), where the sintering of 300 µm thick layers of polyamide powder (DuraForm® PA from 3DSystems™ Inc., Rock Hill, SC, USA). The SLS is one of the most used AM technologies in the industrial framework. It allows for processing thermoplastic materials and is a leading method to produce ready to use objects of highly complex shapes. The CAD-designed OECT consists of a 3D structure made up by three cubes (pads, yellow in Figure 1A), connected to each other at the bottom by a T-shaped feature (white in Figure 1A), responsible for spatial configuration stability. The slicing of the 3D model was executed by the open source software, Slic3r ver. 1.2.9, this operation precedes the actual fabrication process and it is used to generate the G-code for the 3D printer. The SnowWhite SLS was then used with the following parameters to obtain the final object: output power 5.6 W; laser scan rate 6400 pts/s; environment temperature 142 °C. The un-sintered powder was removed and a post-curing process at 140 °C for 15 min was carried out. An Au evaporation was performed in an e-beam ULVAC (EBX-14D) to uniformly cover the pads after an accurate 3D shadow masking of the channel, gate, and T-shaped structures. The structures grown on the cubes are the core part of the OECT device, i.e., the channel (the bridge-like structure) and the gate (the L-shaped structure, blue in Figure 1A), respectively. The spacing between the channel and gate electrodes is 1 mm while the nominal area of the PEDOT:PSS active channel is 1.8 cm^2^ (the length of the upper part of the bridge-like structure is 1.5 cm). The device channel and gate electrode were obtained by dipping the structure in PEDOT:PSS (Clevios PH 1000) pretreated with 1% *v*/*v* of ethylene glycol (EG, for secondary doping) and 0.2% *v*/*v* of 3-glycidoxypropyltrimethoxysilane (GOPS, for improving film stability and adhesion) and, afterwards, the device was annealed at 120 °C for 90 min in a vacuum chamber in order to enhance the PEDOT:PSS conductivity and stability. The procedure was repeated twice.

An optical image of the manufactured device is reported in Figure 1B. The dip coating step was revealed to be a simple and effective method for the integration of the organic active material onto the insulating polyamide 3D structure. Alternative approaches (spray coating, evaporation from Knudsen cells, atomic layer deposition) may, of course, be considered for more complex 3D structures, providing complementary advantages and drawbacks, together with the development of novel 3D printable functional materials [21,22] currently under investigation by the authors of the present paper.

High-resolution scanning electron microscopy has been used to acquire micrographs of the sintered polyamide before (magnification 1500×) and after (magnification 1000×) PEDOT:PSS coating, by means of a field emission gun scanning electron microscope (ZEISS FESEM-FIB Auriga Compact, Oberkochen, Germany). Micrographs have been acquired using an extra high tension of 0.5 kV, at a working distance of 3 mm and vacuum conditions of 3 × 10^−6^ mbar.

The device electrical characterization was carried out with the dual aim of showing the device OECT-like response and sensing capability against the vapor phase generated by water, aqueous solutions, and perspiration.

The OECT response has been evaluated by performing transfer characteristics, i.e., channel current (I_ds_) as a function of the gate-to-source voltage (V_gs_) at a fixed source-to-drain voltage (V_ds_). Specifically, I_ds_ vs V_gs_ curves have been acquired by fixing V_ds_ at −0.2 V and by sweeping V_gs_ between −0.2 and 2 V with a scan step and scan rate of 0.2 V and 10 s, respectively. PBS10X dilution in deionized (DI) water to PBS1X (corresponding to a solution with phosphate buffer concentration of 10^−2^ M) acted as the gate electrolyte. In detail, the bridge-like and L-shaped structures have been both immersed into the gate electrolyte, determining 1.2 cm^2^ and 0.6 cm^2^ of the PEDOT:PSS channel and gate surface areas in contact with the electrolyte, respectively. From the acquired transfer curve, the device transconductance (g_m_), which is the typical figure of merit of transistors that, in the case of OECTs, expresses the attitude at amplifying ionic signals transduced by the PEDOT:PSS channel, has been assessed as g_m_ = |∂I_ds_/∂V_gs_|.

Resistive sensor-like measurements have been performed in real-time mode, by acquiring the I_sens_ time evolution at V_sens_ = −0.3 V using a two-terminal configuration (gate electrode disconnected) at room temperature, in proximity of aqueous surfaces of DI water and PBS dilution in DI water, and by placing the device next to sweaty skin, respectively. In all cases, upon channel biasing and after waiting for the channel current stabilization (a condition satisfied after about 100 s of channel biasing), the device has been manually placed using a manipulator next to the testing surface (water or skin) and then removed in order to check the sensor reversibility. Two different concentrations of phosphate buffer in PBS, i.e., PBS10X and PBS1X, have been used to check the device responsiveness in the presence of a relative lowering of vapor pressure in the case of PBS10X with respect to the diluted solution (PBS1X) that, according to Raoult’s law, is due to the higher mole fraction of salts in the former solution (PBS10X). The distance between the liquid surface and the device during the sensing operation has been fixed at 1 mm for 300 s. Sensitiveness to perspiration has been evaluated by exposing the device next to sweaty skin, again at 1 mm, for 200 s. Control measurements have been acquired by replicating the real-time measurement in the presence of both empty liquid containers and dry skin, respectively. The whole electrical characterization has been carried out using a 2-channel source-meter precision unit (B2902A, Keysight Technologies, Santa Clara, CA, USA).

## 3. Results and Discussion

The recorded device transfer curve reveals a typical OECT response due to the interaction between cationic species in the electrolyte, which are injected upon the application of a positive V_gs_ into the PEDOT:PSS bulk, and oxidized sites on the PEDOT phase. Such interaction leads to the reduction of PEDOT phase resulting in a lowering of I_ds_.

**Figure 2:**
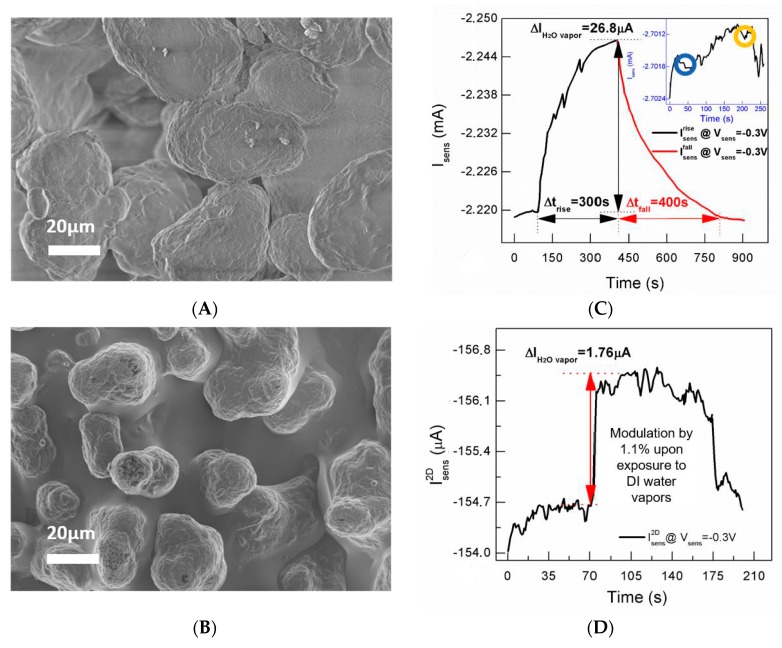
SEM images of (**A**) the sintered polyamide (1500×) and (**B**) the same coated by a poly(3,4-ethylenedioxythiophene):polystyrene sulfonate (PEDOT:PSS) film (1000×); (**C**) real-time I_sens_ change produced by water vaporization at a distance of 1 mm (black branch) and reversibility check (restoring of the initial I_sens_ value upon removal of the water reservoir, red branch). The inset shows the device behavior upon exposure to an empty reservoir and removal of the same reservoir (20 s lasting effects on the recorded current upon reservoir exposure and removal are indicated by the blue-circled and orange-circled regions, respectively); (**D**) comparative real-time measurement carried out on a 2D device inducing a percentage I_sens_ change comparable to that of the 3D sensor: the change is faster but requires a 33% wider active nominal PEDOT:PSS surface area than that of 3D prototype.

In our case, a marked current modulation at lower V_gs_ and a milder trend, tending to a saturating behavior, at higher V_gs_ are exhibited by the device (Figure 1B). The device transconductance peak value (red curve of Figure 1B), ranging around 1 mS and overperforming the values reported in the literature for all-PEDOT:PSS OECT structures [23,24], indicates efficient transduction of ionic signals generated in the electrolytic medium [25]. For all-PEDOT:PSS devices with active channels characterized by a length-to-width ratio greater than 1, the peak values have been found to be lower by at least one order of magnitude [24]. The enhancement of transconductance peak value is reasonably due to the porous nature of the substrate that favors the formation of thicker PEDOT:PSS films [26] realizing, hence, a high surface area-to-volume ratio. In this respect, SEM micrographs of the bare sintered polyamide 3D substrate (Figure 2A) and the same structure coated with PEDOT:PSS (Figure 2B) show that the roughness of the polyamide surface is accordingly transferred to the PEDOT:PSS coating. Even if g_m_ peak enhancement is not an intrinsic consequence of the 3D extent, the combination of the coating technique (dipping) and substrate properties (porosity) allow obtaining defined channels regardless of pattering strategies and superior amplifying performance than that of all-PEDOT:PSS planar (2D) devices.

The higher applied V_gs_ values fall in the range at which water hydrolysis takes place (the reaction has a standard potential of −1.23 V), undesirable for bio-applications due to the arising of faradaic currents. However, in our case the device shows a transconductance peak value and related ion-to-electron transduction effectiveness by the PEDOT:PSS transducer below the electrochemical window of water (i.e., at V_gs_ = 0.6 V), hence the device operation is compatible with biological ambient applications even without recurring to a specific gate electrode functionalization, as reported in the case of different gate materials [27]. Low V_gs_ values in correspondence with the transconductance peak have also been reported in the case of all-PEDOT:PSS 2D devices. This is a typical feature of all-PEDOT:PSS devices that can be reasonably ascribed to the fact that the gate electrode and the device channel both experience a change of their red-ox properties during the measurement upon V_gs_ scan sweep. In our case, no remarkable effect of faradaic reactions, at least below V_gs_ = 1.6 V, is evidenced by the device transconductance curve, showing the onset of a second peak above V_gs_ = 1.6 V (red-circled). Moreover, PEDOT:PSS does not undergo any overoxidation due to protons eventually produced above V_gs_ = 1.6 V. In fact, the device operation has been checked to be reproducible and reversible upon repeated measurements by sweeping the gate voltage in the reported range (−0.2 V, +2 V).

As far as the modulation capability is concerned, the current ON–OFF ratio, calculated from the transfer curve as the ratio between the highest (in our case, at V_gs_ = −0.2 V) and lowest (at V_gs_ = 2 V) I_ds_ values, represents a parameter expressing a measure of the device modulation efficiency. The low ON–OFF ratio of about 2 is because the modulation efficiency of all-PEDOT:PSS OECTs is strongly sensitive to the device specifications in so far as an efficient response requires a high gate electrode/active channel surface ratio (in our case, the gate-to-channel surface ratio is 0.5) [23].

The OECT performance must not be cause for concern. On one hand, 2D OECT structures used for the detection of dopamine have shown similar performances in terms of modulation capability [24]. The device response, in addition, can be modulated and enhanced on-demand by simply modeling the features (shape, size, and distance) of the spacing between the gate terminal and the active channel, also promoting a better coupling between the gate electrode and the active channel. The adopted spacing (1 mm), for instance, was selected just to demonstrate the feasibility of the exposed 3D structuring idea, and, indeed, it is a very relaxed one with respect to the nominal resolution of the employed SLS 3D printer (100 µm).

PEDOT:PSS is notoriously responsive to humidity and the literature shows the use of PEDOT:PSS as the active sensing material in devices dedicated to humidity detection, generally evidenced by an enhancement of the device channel current [20,28]. Two-terminal devices based on PEDOT:PSS/nanosystem composites [29,30] have indicated a larger exposed surface for the composites with respect to that of bare polymers such as those responsible for an enhanced humidity detection.

This observation suggests that the porous substrate and the consequent enhancement of PEDOT:PSS surface area-to-volume ratio may assist the interaction between the device channel and water vapor fluxes. Real-time tests performed by placing the device in proximity of a water surface, using a two-terminal configuration at a fixed channel voltage, reinforce this hypothesis (Figure 2C). Specifically, after an initial instability taking place due to ambient conditions and quantified in the time interval 0 to 100 s (t = 100 s is the time at which the device is exposed to water vapors) as a current increase by 0.03% of its value, the expected channel current increase [20] occurs upon exposing the device next to the surface of a water reservoir for 300 s (black branch). The I_sens_ change at 400 s is by 1.2% with respect to its initial value I_sens_ (t = 100 s). By comparison, a 2D resistive device has a reasonably thinner PEDOT:PSS film (100 nm thick, deposited on a glass substrate by spin coating at 2000 rpm for 30 s) than that of the 3D structure, has provided in similar experimental conditions (V_enss_ = −0.3 V) a I_sens_ enhancement by 1.1% of the initial current value. The change is comparable to that of the 3D device (1.2%) and, though it is faster (due to both a thinner PEDOT:PSS film realizing a faster vapor permeation through the PSS phase and the condensation effect favored by the glass substrate in proximity of the water surface), it requires a greater nominal active area of 2.4 cm^2^ (Figure 2D).

The small I_sens_ change can be rationalized by evaluating the rate of water vaporization (g_hour_,) under the measured experimental ambient conditions. g_hour_ assessment can be carried out using the (empirical) equation:

(1) $$g_{\mathrm{hour}}=E_{C}\times S\times \left(X_{s}-X\right)$$

where E_C_ = 25 + 18 × v ([kg/(m^2^·h)]) is the empirically determined evaporation coefficient (v is the speed of the air above the water surface; in our case we can assume v = 0 m/s), S is the water surface area, X_s_ is the maximum humidity ratio of saturated air at the same temperature as the water surface, and X = 0.622 × p_w_/(p_a_ − p_w_) is the humidity ratio of air (p_w_ and p_a_ are the partial pressure of water vapor in moist air and atmospheric pressure of moist air, respectively [31]). Measured experimental ambient conditions of relative humidity (30 %RH) and T_room_ = 293 K imply a low evaporation rate of about 20 µg/min, corresponding to 60 µg of water vapor produced during the experiment. By considering the volume comprised between the water surface and an ideal plane (parallel to water) containing the device channel interface facing the water, the vapor density between the same water and device interface produced during the sensing operation is 1.2 g/m^3^, corresponding to about 1200 ppm of water vapor, which is the maximum amount of water vapor ideally impacting on the active device surface during the operation. It stands to reason that a small quantity of water vapor interacts with the materials during the exposure, hence the expected very low %RH variation due to water vaporization is compatible with performances of resistive humidity sensors based on PEDOT:PSS and operating in their dynamic range. For instance, a resistivity/conductivity variation by a factor 100 has been assessed upon varying the %RH in a large window, from 55% to 85%, in the case of state-of-the-art resistive humidity sensors based on pristine PEDOT:PSS [28].

The I_sens_ recovery upon removal of the water reservoir, taking place on a time window of 400 s (red branch), certifies the reversibility of such effect. Control experiments indicate that the I_sens_ enhancement is unambiguously ascribable to water vaporization, as confirmed by the practically unchanged signal upon device exposure to an empty water reservoir (see inset Figure 2C).

**Figure 3:**
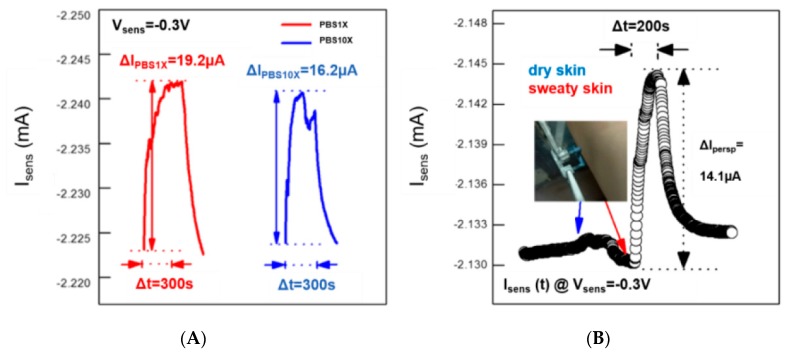
(**A**) Effect of the saline content on the vaporization process of water: red and blue curves represent real-time measurements in the presence of PBS1X and PBS10X, respectively; (**B**) device sensitiveness to perspiration evidenced by a real-time experiment carried out by placing the device at 1 mm from the dry and sweaty skin. Blue and red arrows indicate the positioning of the device next to dry and sweaty skin (200 s of exposure), respectively.

In light of such results, we have also carried out real-time measurements in the presence of two PBS10X and PBS1X in order to show how the device can sense variations of the vapor pressure. This is because the vapor pressure of a solution at different concentrations of non-volatile salts and divalent cations is known to be lower than that of the solvent (DI water for PBS), and the lowering is directly proportional to the mole fraction of the dissolved salts and divalent cations. As reported in Figure 3A, the device is able to monitor the reduction of the vapor pressure (in terms of a less efficient conductivity enhancement after 300 s of exposure) upon increasing the molar fraction of species dissolved in DI water.

The device responsiveness to vapor fluxes suggests that real-time measurements can show how the device can be employed in a real case of interest for medicine. In detail, the device sensing capability in the presence of vapor fluxes produced by perspiration has been evaluated by placing it next to dry and sweaty skin (Figure 3B). The instability of the measurable current in the order of 1 µA (less than 0.05% of the initial I_sens_ value) has been recorded upon 200-second exposure to dry skin (control measurement), while the same duration of exposure to sweaty skin has shown a current enhancement of about 0.7%.

Beyond the demonstrated examples of applications of the 3D prototype, it is noteworthy that the versatility of the proposed fabrication method and its user-friendly character allow us to explore several solutions whose efficiency can be assisted by the three-dimensional extent, in a simple and direct way. Solid electrolytes or materials with specific functionalities, such as reactivity to selected analytes, may be used to fill the spacing between the gate electrode and the active channel with the aim of implementing a selective, spatial sensing operation. We are currently moving towards this direction, also characterizing the response of 3D prototypes with more elaborate geometries than the proposed one.

## 4. Conclusions

In conclusion, we have illustrated the potentialities of an easy and fast manufacturing method suitable to fabricate 3D organic devices based on solution-processable conductors. In particular, we have shown the effectiveness of the method upon fabricating a simple prototype of a 3D device fulfilling the requirements of a multifunctional approach, thanks to the combination of the 3D SLS features and PEDOT:PSS properties. The device has been demonstrated to work as an OECT characterized by high gains compared to those of planar two-dimensional all-PEDOT:PSS OECTs. The device combines the water vapor sensing capability with the intrinsic switching functionality of OECTs. In fact, it is effective in detecting both water evaporation and skin perspiration when operated upon a two-terminal configuration. The sensing effectiveness has been checked upon changing the vapor pressure of water by means of different concentrations of the phosphate buffer in PBS. To our knowledge, this is the first reported proof-of-concept of a true 3D-structured OECT obtained by exploiting a SLS approach. Even if different, more complex or more miniaturized versions and 3D layout may be conceived, the presented prototype paves the way for the conception of a new generation of organic sensing devices that can be structured, through rapid re-design, in a variety of customized 3D implementations for different application areas (biomedical, textile, automotive, and sport and leisure).
